# Synthesis and Evaluation of a Series of Novel Asymmetrical Curcumin Analogs for the Treatment of Inflammation

**DOI:** 10.3390/molecules19067287

**Published:** 2014-06-04

**Authors:** Yali Zhang, Leping Zhao, Jianzhang Wu, Xin Jiang, Lili Dong, Fengli Xu, Peng Zou, Yuanrong Dai, Xiaoou Shan, Shulin Yang, Guang Liang

**Affiliations:** 1 School of Environmental and Biological Engineering, Nanjing University of Science and Technology, Nanjing 210094, Jiangsu, China; E-Mails: ya-li000@163.com (Y.Z.); top_zoupeng@163.com (P.Z.); 2 Chemical Biology Research Center at School of Pharmaceutical Sciences, Wenzhou Medical University, Wenzhou 325035, Zhejiang, China; E-Mails: wujianzhang6@163.com (J.W.); jx334555768@163.com (X.J.); wzmcliangguang@163.com (G.L.); 3 Department of Pharmacy at the Affiliated Yueqing Hospital, Wenzhou Medical University, Wenzhou 325699, Zhejiang, China; E-Mail: leping1967@163.com; 4 The 2nd Affiliated Hospital, Wenzhou Medical University, Wenzhou 325035, Zhejiang, China; E-Mails: luckydlily@163.com (L.D.); 18368719239@163.com (F.X.); yuanrongdai2000@163.com (Y.D.)

**Keywords:** curcumin, asymmetrical curcumin analogs, anti-inflammation, QSAR, cytokines

## Abstract

Curcumin has been reported to possess multiple bioactivities, such as antioxidant, anticancer, and anti-inflammatory properties, however the clinical application of curcumin has been significantly limited by its instability and poor metabolism. Modification of curcumin has led to discovery and development of lots of novel therapeutic candidates. In recent years acute and chronic inflammation has been the focus of numerous studies in various diseases. Here, we synthesized a series of asymmetrical curcumin analogs with high *in vitro* chemical stability, and their anti-inflammatory activity was evaluated in LPS-stimulated macrophages. According to the bio-screening results and QSAR analysis, these analogs exhibited potent activities against LPS-induced TNF-α and IL-6 release. Among the analogs of the potent anti-inflammatory activity, compounds **3b8** and **3b9** exhibited significant protection and possess enhanced anti-inflammatory activity thereby attenuated the LPS-induced septic death in mice.

## 1. Introduction

Curcumin is an active ingredient of the perennial herb *Curcuma longa* (commonly known as turmeric). It has been shown to exhibit antioxidant, anti-inflammatory, antiviral, and antibacterial activities [1]. Thus, curcumin has therapeutic potential against cardiovascular diseases, cancers [2], diabetes [3], allergies [4], arthritis and viral hepatitis [5,6]. Despite the favorable biological properties of curcumin, there are some limitations for the development of curcumin as a potential therapeutic drug, which include its low bioavailability *in vivo* and instability [7,8]. The level of curcumin after its administration has been evaluated by many studies and has been reported to be either negligible or only low in serum or tissue [9,10,11]. In addition to poor bioavailability, curcumin is unstable in aqueous solution at physiological pH and undergoes rapid degradation into molecular fragments [12]. Wang *et al.* found that within 30 min 90% of curcumin was degraded into various products in phosphate buffer (pH 7.4) [13]. Recent studies indicate that the β-diketone moiety appears to be a specific substrate for a series of aldo-keto reductases [14,15]. Therefore, it is hypothesized that the β-diketone moiety contributes to the limited clinical applications of curcumin.

**Scheme 1:**
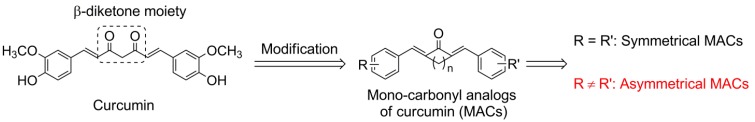
The design of mono-carbonyl curcumin analogs.

In our previous studies, as shown in Scheme 1, we found that several series of symmetrical mono-carbonyl analogs of curcumin lacking the β-diketone moiety exhibited enhanced stability, improved pharmacokinetic profiles [16], and enhanced anti-inflammatory activity both *in vitro* and *in vivo* [17]. However, there are little researches focused on asymmetrical mono-carbonyl curcumin analogs with different constituents on the two phenyl rings and their anti-inflammatory activities [18]. Therefore, as a continuation of our previous studies, we recently directed our attention to the asymmetrical mono-carbonyl curcumin analogs as novel potential anti-inflammatory candidates (Scheme 1).

The inflammatory response plays an important role in the pathological processes of many diseases, including sepsis, cancer, diabetic complications, and cardiovascular disorders [19,20]. During the inflammatory process, different cell types are recruited, including monocytes that locally differentiate into macrophages. This leads to the regulated production of various pro-inflammatory mediators, including Interleukin-6 (IL-6) and tumor necrosis factor-α (TNF-α), both of which play critical roles in promoting the inflammatory response and pathological development [21]. Inhibition of the expression of inflammatory cytokines by small molecules or antagonism of their actions by antibodies has been considered to be effective strategies for the treatment of inflammation-related diseases [22]. Curcumin has been reported to suppress the expression of various inflammatory cytokines, including TNF-α and IL-6 [23]. Previously, we have evaluated the anti-inflammatory properties of symmetrical mono-carbonyl analogs of curcumin using lipopolysaccharide (LPS)-stimulated mouse RAW 264.7 macrophages [16,17]. Here, we present a series of asymmetrical curcumin analogs and test their anti-inflammatory activities in mouse RAW 264.7 macrophages. We also show that these asymmetrical curcumin analogs are considerably more chemically stable than curcumin in pH 7.4 buffer. Accordingly, the active compound, **3b9**, exhibited a beneficial effect in an LPS-induced septic mouse model.

## 2. Results and Discussion

### 2.1. Chemistry

**Scheme 2:**
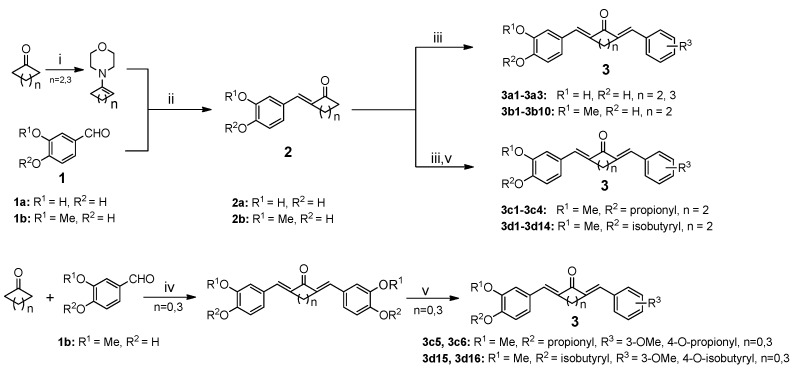
The synthetic routes for the target compounds.

Scheme 2 shows the synthetic routes of the curcumin derivatives, in which the aromatic ring was substituted by various groups. Four symmetrical mono-carbonyl analogs of curcumin, **3c5**, **3c6**, **3d15**, and **3d16**, were prepared for bioactivity comparison and they are synthesized from symmetrical intermediates with propionyl or isobutyryl chloride in the presence of Et_3_N at room temperature, respectively.

*Reagents and conditions*: (**i**) 4-Methylbenzenesulfonic acid, cyclohexane, 90 °C, reflux, 4 h; (**ii**) EtOH, 90 °C, reflux, 4 h, aq. saturated HCl; (**iii**) different aldehydes, HCl gas, EtOH, 50–70 °C, reflux, 3–5 h; (**iv**) HCl gas, EtOH, rt, overnight; (**v**) THF, propionyl or isobutyryl chloride, Et_3_N, rt, overnight.

In the synthesis of asymmetrical analogs, enamine intermediates were used according to the previously reported methods [24]. Commercially available protocatechualdehyde (**1a**) or vanillin (**1b**) reacted with various enamine intermediates in the presence of HCl-saturated EtOH to produce intermediates **2a** or **2b**. Reaction of the **2a** or **2b** with various aromatic aldehydes in the presence of HCl gas afforded compounds **3a1**–**3a3** and **3b1**–**3b10**. Electrophilic aromatic substitution of the OH group with propionyl or isobutyryl chloride in THF at ambient temperature yielded compounds **3c1**–**3c4** and **3d1**–**3d14**. The structures of all compounds are shown in Table 1 and they were characterized using ^1^H-NMR, HPLC, and ESI-MS.

**Table 1:** The chemical structures of the synthetic curcumin analogs. 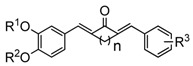

Comp.	R_1_	R_2_	R_3_	n	Comp.	R_1_	R_2_	R_3_	n
**3a1**	H	H	3,4-OMe	3	**3c6**	Me	propionyl	3-OMe, 4-O-propionyl	3
**3a2**	H	H	3,4,5-OMe	2	**3d1**	Me	isobutyryl	2-F	2
**3a3**	H	H	3,4,5-OMe	3	**3d2**	Me	isobutyryl	4-F	2
**3b1**	Me	H	2-F	2	**3d3**	Me	isobutyryl	2-OMe	2
**3b2**	Me	H	4-F	2	**3d4**	Me	isobutyryl	4-F	2
**3b3**	Me	H	2-Cl	2	**3d5**	Me	isobutyryl	4-OEt	2
**3b4**	Me	H	4- *tert*-butyl	2	**3d6**	Me	isobutyryl	3,4-OMe	2
**3b5**	Me	H	2-OMe	2	**3d7**	Me	isobutyryl	2,5-OMe	2
**3b6**	Me	H	3-OH, 4-OMe	2	**3d8**	Me	isobutyryl	3,4,5-OMe	2
**3b7**	Me	H	4- *di*-ethylamino	2	**3d9**	Me	isobutyryl	2-O-isobutyryl	2
**3b8**	Me	H	4- *di*-butylamino	2	**3d10**	Me	isobutyryl	4-O-isobutyryl	2
**3b9**	Me	H	4-piperidine	2	**3d11**	Me	isobutyryl	3-O-isobutyryl, 4-OMe	2
**3b10**	Me	H	4-morpholine	2	**3d12**	Me	isobutyryl	3,4-O-isobutyryl	2
**3c1**	Me	H	2-F	2	**3d13**	Me	isobutyryl	4- *di*-ethylamino	2
**3c2**	Me	propionyl	4- *di*-ethylamino	2	**3d14**	Me	isobutyryl	4-morpholine	2
**3c3**	Me	propionyl	4-piperidine	2	**3d15**	Me	isobutyryl	3-OMe,4-O-isobutyryl	0
**3c4**	Me	propionyl	4-morpholine	2	**3d16**	Me	isobutyryl	3-OMe,4-O-isobutyryl	3
**3c5**	Me	propionyl	3-OMe, 4-O-propionyl	2					

### 2.2. Biological Evaluation

#### 2.2.1. Anti-Inflammatory Activity

Lipopolysaccharide (LPS) can stimulate a number of host cells to produce and release a wide variety of proinflammatory cytokines, including TNF-α, IL-1β, IL-6 and IL-8 [25]. The anti-inflammatory abilities of curcumin and its 35 analogs were characterized by evaluating their inhibition against LPS-induced TNF-α and IL-6 release in the mouse macrophages. RAW 264.7 macrophages were pretreated with the compounds for 2 h and then stimulated with LPS for 22 h. ELISA assay was used to detect the quantities of TNF-α and IL-6 in the media and the protein concentration of cells harvested from the same cultural plates was used to normalize the cytokines level.

**Figure 1:**
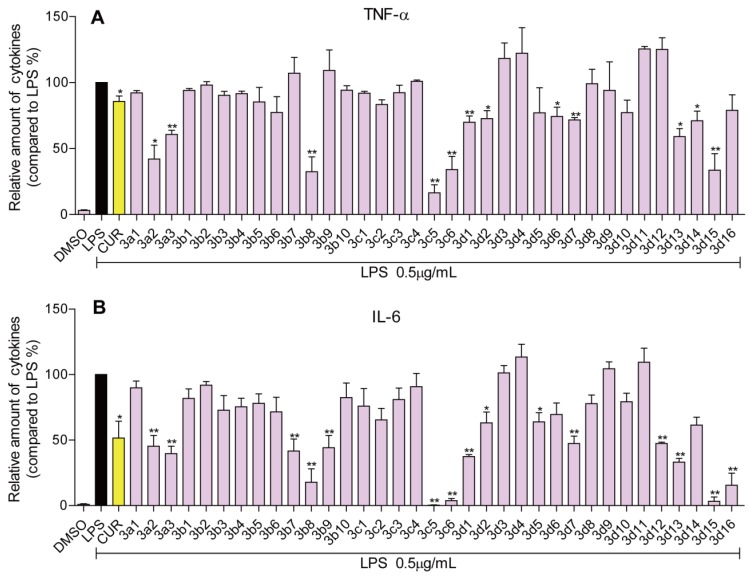
Effects of curcumin and its analogues on TNF-α (**A**) and IL-6 (**B**) production. Macrophages were pre-treated with vehicles, curcumin or its analogues (10 μM) for 2 h, and then treated with or without LPS (0.5 μg/mL) for 22 h. TNF-αand IL-6 levels in the culture media were measured by ELISA and were normalized by the total protein. Values represent the mean ± SEM for three independent experiments and LPS is regarded as 100%. Statistical significance relative to LPS was indicated, *p < 0.05; **p < 0.01.

The results of their inhibitory activities are shown in Figure 1. We found that the majority of the curcumin analogs showed better inhibitory abilities than that of curcumin at a concentration of 10 μM. Among the 35 compounds, 15 showed better inhibitory effects than curcumin on LPS-induced TNF-α expression, and 13 were found to be more potent than curcumin with respect to LPS-induced IL-6 expression.

Overall, the suppression of these compounds toward IL-6 is better than toward TNF-α. All the four symmetrical compounds **3c5**, **3c6**, **3d15**, and **3d16** showed excellent anti-inflammatory activity, and they were found to be more active than the corresponding asymmetrical compounds with the same substituent in one benzene ring, indicating a symmetrical structure is favorable to the anti-inflammatory ability of curcumin analog. Compounds **3b8**, **3c5**, **3c6**, and **3d15** exhibited stronger inhibitory activity against both TNF-α and IL-6 than curcumin, whereas **3b9** and **3d13** only showed stronger inhibitory activity against IL-6 than curcumin. The symmetrical curcumin analogs **3c5** and **3c6** exhibited the strongest inhibitory effects on LPS-induced TNF-α and IL-6 excretion among all of the tested compounds. According to the bio-screening results in asymmetrical analogs, R^3^ being a large N-substituted group (such as piperidine or dibutylamino) and R^2^ being H are beneficial for the bioactivities of these analogs. Simultaneously, compounds with R^1^R^2^R^3^ being lipophilic groups have much stronger anti-inflammatory activity than compounds with one or two lipophilic groups. The large substituent assesses good activities than small substituent according to **3d1** *vs.***3b1** and **3c1**, **3d13** *vs.***3c2** and **3b7**, **3d14** *vs.***3c4** and **3b10**, but not all the same. Compound **3b9** has much stronger inhibitory activity than **3c3**. More curcumin analogs need to be designed and synthesized to explore the detailed structure-activity relationships.

#### 2.2.2. Quantitative Structure-Activity Relationships (QSARs)

**Figure 2:**
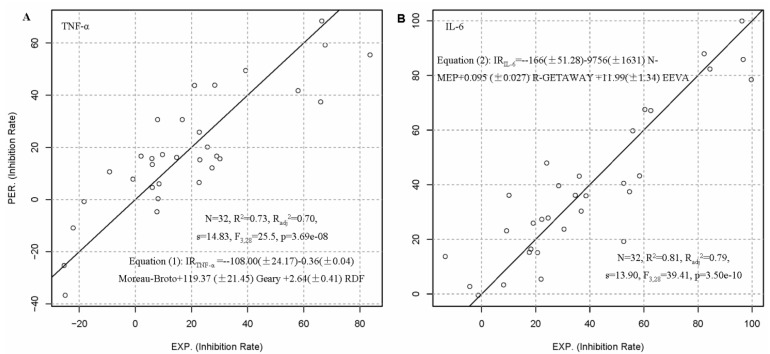
Plot of predicted activity against the corresponding experimental activity on TNF-α (**A**) and IL-6 (**B**) inhibition. N, the number of compounds taken into account in the regression; R^2^, the multiple correlation coefficient; R^2^_adj_, adjusted multiple correlation coefficient; s, residual standard error; and the F value is related to the F-statistic analysis (Fischer test). The numbers in parentheses mean the standard deviation of the coefficients.

To further demonstrate the SARs of these curcumin analogs, we constructed a QSAR model. In general, compounds are often represented by molecular descriptors [26]. These descriptors include quantum-chemical descriptors, topological descriptors, and charge descriptors, among others. We used IR_TNF-α_ and IR_IL-6_ as the factors of their biological activities; the specific descriptors for the QSAR study and a scatter plot of the predicted *vs.* experimental values are shown in Figure 2.

A relatively high regression coefficient (R^2^) of 0.81 for the activities of derivatives for anti-IL-6 was obtained from the data in Figure 2, while the IR_TNF-α_ is of modest quality (R^2^ = 0.73). The variables used in these equations might explain the variance in the IL-6- and TNF-α-inhibitory activities of the curcumin analogs. The QSAR results indicate that the inhibition rate is highly related to the skeleton structure and special substituent in the compounds. Taken together, the SAR and QSAR results on the anti-inflammatory activities of these curcumin analogs may provide valuable information for the further design of novel anti-inflammatory agents.

#### 2.2.3. Six Active Compounds Inhibit TNF-α and IL-6 Release in a Dose-Dependent Manner

**Figure 3:**
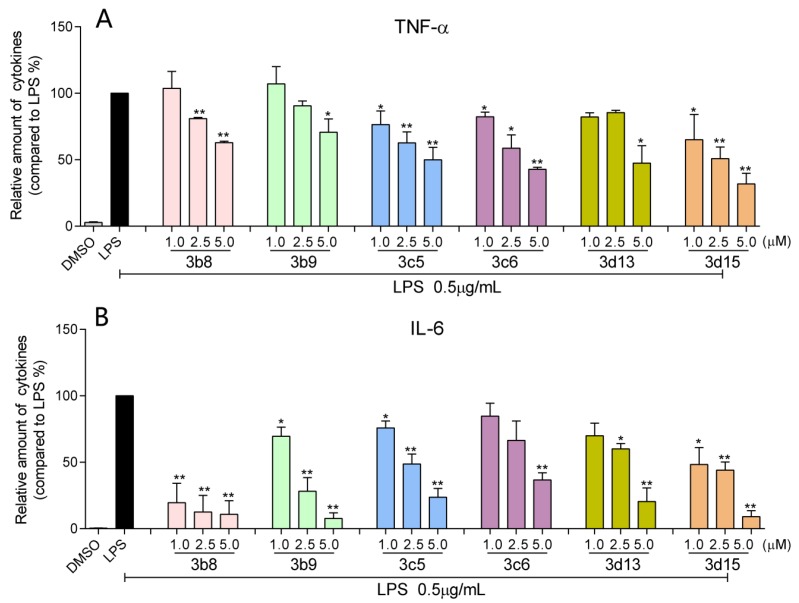
Effects of active compounds on TNF-α (**A**) and IL-6 (**B**) production at dose-dependent manner. Macrophages were treated with vehicle (DMSO) or various concentrations of active compounds for 2 h, then incubated with or without LPS (0.5 µg/mL) for 22 h. TNF-αand IL-6 levels in the culture media were measured by ELISA and were normalized by the total protein. Values represent the mean ± SEM for three independent experiments and LPS is regarded as 100%. Statistical significance relative to LPS was indicated, *p < 0.05; **p < 0.01.

Further, we selected six active compounds **3b8**, **3b9**, **3c5**, **3c6**, **3d13**, and **3d15** to evaluate their inhibitory activity against LPS-stimulated TNF-α and IL-6 secretion in a dose-dependent manner. RAW 264.7 macrophages were pretreated with different concentrations (1.0, 2.5 and 5.0 μM) of the six active analogs for 2 h and were subsequently incubated with LPS (0.5 μg/mL) for 22 h. As shown in Figure 3, these compounds exhibited a dose-dependent inhibition activity against LPS-induced TNF-α and IL-6 release. Compounds **3b8** and **3b9** showed the relatively higher activity than others at the low concentrations (1.0 and 2.5 μM). These results further validated that these compounds might act as a potent anti-inflammatory agent.

#### 2.2.4. The Active Compounds Showed Much Higher Chemical Stability than Curcumin

**Figure 4:**
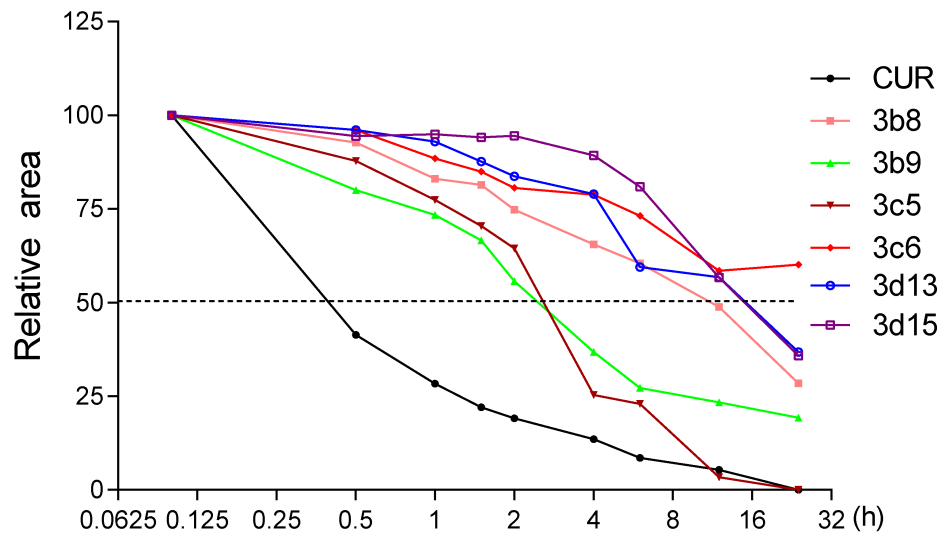
The stability analysis of curcumin and its analogs by HPLC.

Instability and poor metabolism have significantly limited the clinical applications of curcumin. The mono-carbonyl analogs were designed to improve the chemical stability of curcumin. Therefore, we tested the chemical stabilities of six active compounds, **3b8**, **3b9**, **3c5**, **3c6**, **3d13** and **3d15**, using HPLC method in phosphate buffer (pH 7.4) containing 2% methanol at 37 °C. The HPLC figures of compounds at initial time are shown in the Supporting Information. Figure 4 shows the kinetic profiles of curcumin and six analogs during 0–24 h after incubation. Within 30-min of incubation in phosphate buffer, curcumin lost more than 50% of its original peak area and more than 90% during 6 h. However, as can be seen from the results, all the tested substances were reasonably stable in pH 7.4 buffer, with an observed half life > 2 h. Among them, **3b8**, **3c6**, **3d13**, and **3d15** retained 50% of the original peak area, even after 12-h of incubation. We also detected the stabilities of curcumin and its analogs using an absorption spectrum assay, which is described in the Supporting Information. As a result of incubation with phosphate buffer, within 25 min curcumin lost more than 50% of its initial intensity, whereas all of the six tested compounds degraded considerably less than curcumin (Figure S7). This result indicates that these asymmetrical mono-carbonyl curcumin analogs are considerably more stable than curcumin *in vitro*.

#### 2.2.5. Cell Toxicity Assay

**Figure 5:**
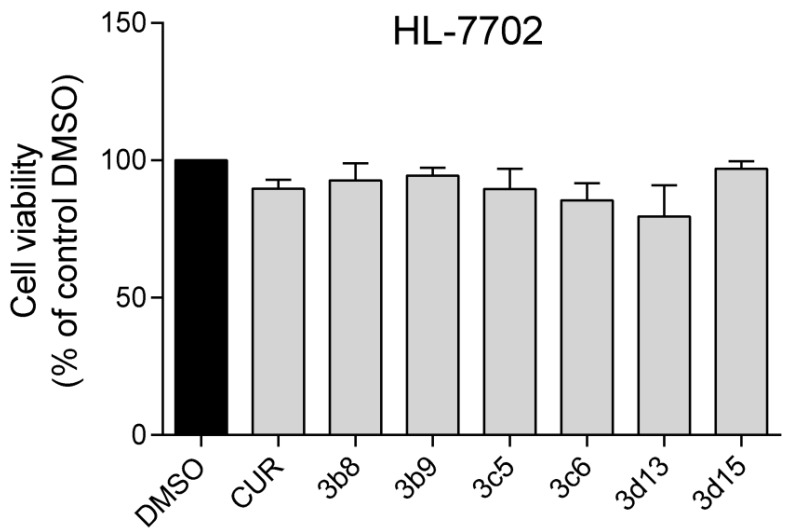
Effects of active compounds on human normal liver HL-7702 cells viability using MTT assay. HL-7702 cells were treated with 10 µM active compounds for 24 h.

Before the biological evaluation *in vivo*, we tested six active compounds, including **3b8**, **3b9**, **3c5**, **3c6**, **3d13** and **3d15**, for their cytotoxicity in the human normal hepatic cell line HL-7702. Cell viability was measured using the MTT method after treatment with the compounds for 24 h. As shown in Figure 5, all of the compounds that were tested exhibited much lower toxicity at a concentration of 10 μM. These effects corresponded to that of curcumin at the same concentration. All of these data indicated that the active compounds are relatively safe. Thus, combining the anti-inflammatory activities and cytotoxic analysis of asymmetrical curcumin analogs, we selected the representative compound **3b8** and **3b9** for the next evaluation *in vivo*.

#### 2.2.6. **3b8** and **3b9** Attenuated the LPS-Induced Septic Death in Mice

As a major endotoxin, LPS has been implicated as a primary cause of sepsis. The evidence that pretreatment of mice with curcumin suppresses the hepatic inflammatory response caused by endotoxin, which indicates the therapeutic effect of curcumin on sepsis and septic shock [27].

**Figure 6:**
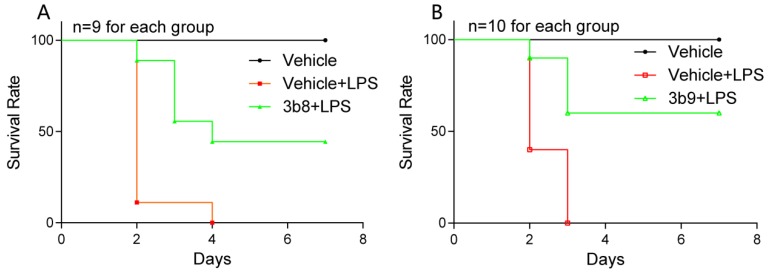
Compounds **3b8** and **3b9** improve survival of mice subjected to a lethal dose of LPS. Mice were intraperitoneal injected with 10 mg/kg **3b8** or **3b9** 15 min before the injection of LPS 25 mg/kg). Survival rates were recorded for 7 days after the LPS injection at the interval of 1 day.

Here, we further determined whether two representative compounds **3b8** and **3b9** are able to attenuate LPS-induced septic death in mice. The compounds **3b8** and **3b9** were administered in a water-soluble form for intravenous injection. Mice were injected intravenously with the compounds **3b8** and **3b9** at a dosage of 10 mg/kg, after 15 min LPS at a dosage of 25 mg/kg was injected intraperitoneally and then the survival rates were monitored for 7 days. As shown in Figure 6, 100% of the animals treated with LPS alone died within 4 days because of septic shock. In animals that received **3b8** and **3b9**, the survival rates were increased significantly compared to the LPS treated group. Pretreatment with **3b9** led to a 60% survival. These data support the anti-inflammatory effects of these curcumin analogs *in vivo*.

## 3. Experimental

### 3.1. General Information

All of the reagents for the synthesis were obtained from Sigma-Aldrich (St Louis, Missouri, USA) and Aladdin (Shanghai, China). Thin-layer chromatography (TLC) was performed on Kieselgel 60 F_254,365_ plates. Melting points were determined on a Fisher-Johns melting apparatus and were uncorrected. ^1^H-NMR spectra were recorded on a Bruker 600 MHz instrument. The chemical shifts were expressed as parts per million with TMS as the internal reference. Electrospray ionization mass spectra (ESI-MS) data were recorded in positive mode on a Bruker Esquire 3000^+^ spectrometer. Column chromatography purifications were conducted on Silica Gel 60 (70–230 mesh, E. Merck, Darmstadt, Germany). The purity of all compounds was detected by HPLC (column: Agilent Eclipse XDB C18 5 μm 4.6 mm × 150 mm, flow: 1 mL/min, UV: 430 nm, methanol/water: condition A: 40/60 to 90/10, condition B: 65/35 to 95/5) before doing biological experiments.

### 3.2. Chemical Synthesis

#### 3.2.1. Synthesis of **2a** and **2b**

A mixture of cyclopentanone or hexanone (0.020 mol), 4-methylbenzenesulfonic acid (200 mg), morpholine (0.030 mol) and cyclohexane (20 mL) were refluxed at 90 °C for 4 h. The solvent was then removed under reduced pressure to afford various enamine intermediates as viscous yellow oils. Then, the enamine intermediates were reacted with **1a** or **1b** (10 mmol) in the presence of dilute HCl to obtain the crude products **2a** or **2b**, and the residues were purified by silica gel chromatography.

#### 3.2.2. Synthesis of **3a1**–**3a3**, and **3b1**–**3b10**

A solution of **2a** or **2b** (0.002 mol) and various aromatic aldehydes (0.002 mol) in EtOH (25 mL) was stirred at room temperature. Subsequently, HCl (gas) was bubbled into the mixture for 30 min, and the resulting reaction solution was stirred for 4 h at 90 °C. The mixture was then poured into ice water. A colored precipitate formed was collected by filtration and then purified to afford **3a1**–**3a3**, and **3b1**–**3b10**.

*(2E,6E)-2-(3,4-Dihydroxybenzylidene)-6-(3,4-dimethoxybenzylidene)cyclohexanone* (**3a1**): Yellow powder, 63.2% yield, mp 135.4–138.8 °C, HPLC purity 99.9% (condition A, r.t. 15.898 min). ^1^H-NMR (CDCl_3_) δ: 7.372 (s, 2H, *β*-H, *β*'-H), 7.223 (d, *J* = 1.2 Hz, 1H, H-2'), 7.175 (dd, *J* = 1.2, 8.4 Hz, 2H, H-2, H-6'), 7.121 (d, *J* = 8.4 Hz, 1H, H-6), 6.935 (d, *J* = 8.4 Hz, 2H, H-5, H-5'), 3.837 (s, 6H, 3',4'-OCH_3_), 2.812 (s, 4H, 3''-CH_2_, 5''-CH_2_), 1.598–1.610 (m, 2H, 4''-CH_2_). ESI-MS *m/z*: 367.1 (M+1)^+^, calcd for C_22_H_22_O_5_: 366.1.

*(2E,5E)-2-(3,4-Dihydroxybenzylidene)-5-(3,4,5-trimethoxybenzylidene)cyclopentanone* (**3a2**): Black green powder, 62.5% yield, mp 112.9–115.4 °C, HPLC purity 99.731% (condition A, r.t. 14.740 min). ^1^H-NMR (DMSO-*d*_6_) δ: 7.365 (s, 1H, *β*-H), 7.292 (s, 1H, *β*'-H), 7.118 (d, *J* = 1.2 Hz, 1H, H-2), 7.027 (d, *J* = 8.4 Hz, 1H, H-6), 6.995 (s, 2H, H-2', H-6'), 6.846 (d, *J* = 7.8 Hz, 1H, H-5), 3.731 (s, 9H, 3',4',5'-OCH_3_), 3.010–3.136 (m, 4H, 3''-CH_2_, 4''-CH_2_). ESI-MS *m/z*: 382.9 (M+1)^+^, calcd for C_22_H_22_O_6_: 382.1.

*(2E,6E)-2-[(3,4-Dihydroxyphenyl)methylidene]-6-[(3,4,5-trimethoxyphenyl)methylidene]cyclohexan-1-one* (**3a3**): Yellow powder, 71.4% yield, mp 135.4-138.8 °C, HPLC purity 92.476% (condition A, r.t. 19.925 min). ^1^H-NMR (CDCl_3_) δ: 7.753 (s, 2H, *β*-H, *β*'-H), 7.260 (s, 1H, H-2), 7.113 (dd, *J* = 1.2, 8.4 Hz, 2H, H-5, H-6), 6.909 (d, *J* = 8.4Hz, 2H, H-2', H-6'), 3.916 (s, 9H, 3',4',5'-OCH_3_), 2.938-2.958 (m, 4H, 3''-CH_2_, 5''-CH_2_), 1.818-1.830 (m, 2H, 4''-CH2). ESI-MS *m/z*: 397.0 (M+1)^+^, calcd for C_23_H_24_O_6_: 396.2.

*(2E,5E)-2-[(2-Fluorophenyl)methylidene]-5-[(4-hydroxy-3-methoxyphenyl)methylidene]cycopentan-1-one* (**3b1**): Black green powder, 50.2% yield, mp 169.6–173.2 °C, HPLC purity 98.982% (condition A, r.t. 18.932 min). ^1^H-NMR (DMSO-*d*_6_) δ: 7.752 (t, *J* = 15.0 Hz, 1H, *β*'-H), 7.493 (t, *J* = 6.6 Hz, 1H, H-4'), 7.433 (s, 1H, *β*-H), 7.339 (d, *J* = 7.8 Hz, 2H, H-3', H-6'), 7.267 (s, 1H, H-5'), 7.186 (t, *J* = 7.8 Hz, 2H, H-2, H-6), 6.900 (d, *J* = 8.4 Hz, 1H, H-5), 3.327 (s, 3H, 3-OCH_3_), 3.070 (s, 4H, 3''-CH_2_, 4''-CH_2_). ESI-MS *m/z*: 325.0 (M+1)^+^, calcd for C_20_H_17_FO_3_: 324.1.

*(2E,5E)-2-[(4-Fluorophenyl)methylidene]-5-[(4-hydroxy-3-methoxyphenyl)methylidene]cyclopenan-1-one* (**3b2**): Orange yellow powder, 35.8% yield, mp 179.5–183.7 °C, HPLC purity 98.744% (condition A, r.t. 19.210 min). ^1^H-NMR (DMSO-*d*_6_) δ: 7.747 (t, *J* = 8.4 Hz, 2H, H-2', H-6'), 7.407 (s, 2H, *β*-H, *β*'-H), 7.325 (t, *J* = 8.4 Hz, 2H, H-3', H-5'), 7.261 (d, *J* = 1.8 Hz, 1H, H-2), 7.092 (dd, *J* = 1.8, 8.4 Hz, 1H, H-6), 6.896 (d, *J* = 8.4 Hz, 1H, H-5), 3.839 (s, 3H, 3-OCH_3_), 3.071 (s, 4H, 3''-CH_2_, 4''-CH_2_). ESI-MS *m/z*: 324.9 (M+1)^+^, calcd for C_20_H_17_FO_3_: 324.1.

*(2E,5E)-2-[(2-Chlorophenyl)methylidene]-5-[(4-hydroxy-3-methoxyphenyl)methylidene]cyclopeantan-1-one* (**3b3**): Orange yellow powder, 49.8% yield, mp 169.4–171.3 °C, HPLC purity 93.977% (condition A, r.t. 20.260 min). ^1^H-NMR (CDCl_3_) δ: 7.892 (s, 1H, *β*'-H), 7.575 (t, *J* = 8.4 Hz, 2H, H-3', *β*-H), 7.460 (dd, *J* = 1.2, 7.8 Hz, 1H, H-6'), 7.279–7.327 (m, 2H, H-4', H-5'), 7.201 (d, *J* = 1.8 Hz, 1H, H-2), 7.093 (dd, *J* = 1.2, 7.8 Hz, 1H, H-6), 6.989 (d, *J* = 7.8 Hz, 1H, H-5), 3.950 (s, 3H, 3-OCH_3_), 3.059–3.080 (m, 4H, 3''-CH2, 4''-CH2). ESI-MS *m/z*: 340.8 (M+1)^+^, calcd for C_20_H_17_ClO_3_: 340.1.

*(2E,5E)-2-[(4-Tert-butylphenyl)methylidene]-5-[(4-hydroxy-3-methoxyphenyl)methylidene]cyclopean-tan-1-one* (**3b4**): Yellow powder, 37.5% yield, mp 177.3–179.6 °C, HPLC purity 99.941% (condition B, r.t. 16.332 min). ^1^H-NMR (DMSO-*d*_6_) δ: 7.620 (d, *J* = 8.4 Hz, 2H, H-2', H-6'), 7.507 (s, 2H, *β*-H, *β*'-H), 7.389 (d, *J* = 8.4 Hz, 2H, H-3', H-5'), 7.259 (d, *J* = 1.8 Hz, 1H, H-2), 7.179 (dd, *J* = 1.2, 8.4 Hz, 1H, H-6), 6.894 (d, *J* = 8.4 Hz, 1H, H-5), 3.840 (s, 3H, 3-OCH_3_), 3.074 (s, 4H, 3''-CH_2_, 4''-CH_2_), 1.299 (s, 9H, -C(CH_3_)_3_). ESI-MS *m/z*: 362.9 (M+1)^+^, calcd for C_24_H_26_O_3_: 362.2.

*(2E,5E)-2-[(4-Hydroxy-3-methoxyphenyl)methylidene]-5-[(2-methoxyphenyl)methyllidene]cyclopean-tan-1-one* (**3b5**): Orange yellow powder, 47.9% yield, mp 174.5–177.7 °C, HPLC purity 99.827% (condition A, r.t. 18.929 min). ^1^H-NMR (DMSO-*d*_6_) δ: 7.742 (s, 1H, *β*'-H), 7.611 (d, *J* = 6.6 Hz, 1H, H-6'), 7.409 (t, *J* = 8.4 Hz, 1H, H-4'), 7.391 (s, 1H, *β*-H), 7.252 (d, *J* = 1.8 Hz, 1H, H-2), 7.173 (dd, *J* = 1.2, 8.4 Hz, 1H, H-6), 7.108 (d, *J* = 8.4 Hz, 1H, H-5), 7.054 (t, *J* = 7.8 Hz, 1H, H-5'), 6.891 (d, *J* = 8.4 Hz, 1H, H-3'), 3.873 (s, 3H, 2'-OCH_3_), 3.837 (s, 3H, 3-OCH_3_), 3.036 (s, 4H, 3''-CH_2_, 4''-CH_2_). ESI-MS *m/z*: 337.0 (M+1)^+^, calcd for C_21_H_20_O_4_: 336.1.

*(2E,5E)-2-[(4-Hydroxy-3-methoxyphenyl)methylidene]-5-[(3-hydroxy-4-methoxyphenyl)methylidene]-cyclopeantan-1-one* (**3b6**): Orange yellow powder, 38.3% yield, mp 210.4–213.9 °C, HPLC purity 91.032% (condition A, r.t. 14.256 min). ^1^H-NMR (DMSO-*d*_6_) δ: 7.333 (s, 1H, *β*'-H), 7.263 (s, 1H, *β*-H), 7.130(s, 1H, H-2'), 7.090 (t, *J* = 9.0 Hz, 2H, H-6', H-2), 7.049 (d, *J* = 8.4 Hz, 1H, H-6), 6.928 (d, *J* = 8.4 Hz, 1H, H-5), 6.855 (d, *J* = 8.4 Hz, 1H, H-5'), 3.839 (s, 3H, 4'-OCH_3_), 3.828 (s, 3H, 3-OCH_3_), 3.027 (s, 4H, 3''-CH_2_, 4''-CH_2_). ESI-MS *m/z*: 353.0 (M+1)^+^, calcd for C_21_H_20_O_5_: 352.1.

*(2E,5E)-2-[4-(Diethylamino)benzylidene]-5-(4-hydroxy-3-methoxybenzylidene)cyclopeantanone* (**3b7**): Purple powder, 54.1% yield, mp 190.9–194.1 °C, HPLC purity 94.249% (condition A r.t. 23.146 min). ^1^H-NMR (DMSO-*d*_6_) δ: 7.557 (s, 1H, *β*'-H), 7.518 (d, *J* = 8.4 Hz, 2H, H-2', H-6'), 7.487 (s, 1H, *β*-H), 7.198 (dd, *J* = 1.2, 8.4 Hz, 1H, H-6), 7.106 (s, 1H, H-2), 6.978 (d, *J* = 8.4 Hz, 1H, H-5), 6.702 (d, *J* = 7.2 Hz, 2H, H-3', H-5'), 3.947 (s, 3H, 3-OCH_3_), 3.422 (q, *J* = 6.6 Hz, 4H, -CH_2_-N-CH_2_-), 3.079 (s, 4H, 3''-CH_2_, 4''-CH_2_), 1.215 (t, *J* = 7.2 Hz, 6H, -CH_3_ × 2). ESI-MS *m/z*: 378.5 (M+1)^+^, calcd for C_24_H_27_NO_3_: 377.2.

*(2E,5E)-2-[4-(Dibutylamino)benzylidene]-5-(4-hydroxy-3-methoxybenzylidene)cyclopeantanone* (**3b8**): Purple powder, 24.6% yield, mp 148.8–151.5 °C, HPLC purity 98.8875% (condition A, r.t. 19.160 min). ^1^H-NMR (DMSO-*d*_6_) δ: 7.551 (s, 1H, β'-H ), 7.510 (d, *J* = 7.8 Hz, 2H, H-2', H-6'), 7.488 (s, 1H, *β*-H), 7.198 (dd, *J* = 1.8, 8.4 Hz, 1H, H-6), 7.102 (d, *J* = 1.2 Hz, 1H, H-2), 6.979 (d, *J* = 8.4 Hz, 1H, H-5), 6.670 (s, 2H, H-3', H-5'), 5.884 (s, 1H, -OH), 3.946 (s, 3H, 3-OCH_3_,), 3.327 (t, *J* = 7.2 Hz, 4H, -CH_2_-N-CH_2_-), 3.076 (s, 4H, 3''-CH_2_, 4''-CH_2_), 1.596 (s, 4H, -C*H_2_*CH_2_CH_3_ × 2), 1.352–1.389 (m, 4H, -CH_2_C*H_2_*CH_3_ × 2), 0.966 (t, *J* = 7.2 Hz, 6H, -CH_2_CH_2_C*H_3_* × 2). ESI-MS m/z: 434.0 (M+1)^+^, calcd for C_28_H_35_NO_3_: 433.3.

*(2E,5E)-2-(4-Hydroxy-3-methoxybenzylidene)-5-[4-(piperidin-1-yl)benzylidene]cyclopeantanone* (**3b9**): Orange powder, 66.5% yield, mp 218.0–221.1 °C, HPLC purity 91.8411% (condition A r.t. 7.865 min). ^1^H-NMR (DMSO-*d*_6_) δ: 7.541 (s, 1H, *β*'-H), 7.522 (d, *J* = 8.4 Hz, 2H, H-2', H-6'), 7.501 (s, 1H, *β*-H), 7.260 (s, 2H, H-3', H-5'), 7.196 (d, *J* = 7.8 Hz, 1H, H-6), 7.101 (s, 1H, H-2), 6.981 (d, *J* = 7.8 Hz, 1H, H-5), 3.945 (s, 3H, 3-OCH_3_), 3.321 (s, 4H, -CH_2_-N-CH_2_-), 3.079 (s, 4H, 3''-CH_2_, 4''-CH_2_), 1.595–1.701 (m, 6H, -CH_2_-CH_2_-CH_2_-). ESI-MS *m/z*: 390.7 (M+1)^+^, calcd for C_25_H_27_NO_3_: 389.2.

*(2E,5E)-2-[(4-Hydroxy-3-methoxyphenyl)methylidene]-5-{[4-(morpholin-4-yl)phenyl]methyllidene} cyclopentan-1-one* (**3b10**): Brown powder, 37.3% yield, mp >300 °C. ^1^H-NMR (DMSO-*d*_6_) δ: 7.485 (d, *J* = 7.8 Hz, 2H, H-2', H-6'), 7.317 (s, 2H, *β*-H, *β*'-H), 7.117 (s, 1H, H-2), 7.085 (dd, *J* = 1.8, 7.8 Hz, 1H, H-6), 6.935 (d, *J* = 7.8 Hz, 2H, H-3', H-5'), 6.850 (d, *J* = 7.2 Hz, 1H, H-5), 3.835 (s, 3H, 3-OCH_3_), 3.745 (s, 4H, -CH_2_-O-CH_2_-), 3.214 (s, 4H, -CH_2_-N-CH_2_-), 3.026 (s, 4H, 3''-CH_2_, 4''-CH_2_). ESI-MS *m/z*: 391.9 (M+1)^+^, calcd for C_24_H_25_NO_4_: 391.2.

#### 3.2.3. Synthesis of **3c1**–**3c4** and **3d1**–**3d14**

Compounds **3b1**–**3b10** (0.001 mol) and Et_3_N (0.25 mL) were dissolved in THF (10 mL) and cooled to 0 °C. A solution of propionyl or isobutyryl chloride (0.001 mol) was added drop wise into the THF solution. The reaction mixture was stirred at room temperature for overnight. TLC showed that the reaction has been completed. Then the solvent was removed, and the resultant residue was chromatographed over silica gel, eluting with petroleum ether and ethyl acetate to afford the desired products **3c1**–**3c4** and **3d1**–**3d14**. 

*4-{(E)-[(E)-3-(2-Fluorobenzylidene)-2-oxocyclopentylidene]methyl}-2-methoxyphenylpropionate* (**3c1**): Royal yellow powder, 15.3% yield, mp 150.8–153.2 °C. ^1^H-NMR (CDCl_3_) δ: 7.808 (s, 1H, *β*'-H), 7.563–7.600 (m, 2H, *β*-H, H-4'), 7.366 (q, *J* = 7.2 Hz, 1H, H-6'), 7.206 (t, *J* = 7.8 Hz, 2H, H-2, H-6), 7.158 (d, *J* = 8.4 Hz, 1H, H-3'), 7.127 (d, *J* = 9.0 Hz, 1H, H-5'), 7.113 (d, *J* = 8.4 Hz, 1H, H-5), 3.875 (s, 3H, 3-OCH_3_), 3.062–3.106 (m, 4H, 3''-CH_2_, 4''-CH_2_), 2.636 (q, *J* = 7.2 Hz, 2H, -COC*H_2_*CH_3_), 1.284 (t, *J* = 7.2 Hz, 3H, -COCH_2_C*H_3_*). ESI-MS *m/z*: 381.3 (M+1)^+^, calcd for C_23_H_21_FO_4_: 380.1.

*4-{(E)-{(E)-3-[4-(Diethylamino)benzylidene]-2-oxocyclopentylidene}methyl}-2-methoxyphenylpropionate* (**3c2**): Purple powder, 38.1% yield, mp 88.2–90.9 °C, HPLC purity 89.991% (condition B, r.t. 17.423 min). ^1^H-NMR (CDCl_3_) δ: 7.522 (d, *J* = 8.4 Hz, 2H, H-2', H-6'), 7.261 (s, 2H, *β*-H, *β*'-H), 7.209 (d, *J* = 8.4 Hz, 1H, H-6), 7.172 (s, 1H, H-2), 7.085 (d, *J* = 8.4 Hz, 1H, H-5), 6.704 (t, *J* = 5.4 Hz, 2H, H-3', H-5'), 3.873 (s, 3H, 3-OCH_3,_), 3.428 (q, *J* = 6.6 Hz, 4H, -CH_2_-N-CH_2_-), 3.086 (s, 4H, 3''-CH_2_, 4''-CH_2_), 2.633 (q, *J* = 7.8 Hz, 2H, -COC*H_2_*CH_3_), 1.287 (t, *J* = 7.8 Hz, 3H, -COCH_2_C*H_3_*), 1.212 (t, *J* = 7.2 Hz, 6H, -NCH_2_C*H_3_* × 2). ESI-MS *m/z*: 434.9 (M+1)^+^, calcd for C_27_H_31_NO_4_: 433.2.

*2-Methoxy-4-((E)-((E)-2-oxo-3-(4-(piperidin-1-yl)benzylidene)cyclopentylidene)methyl)phenylpropionate*(**3c3**): Purple powder, 37.8% yield, mp 154.7–157.3 °C. ^1^H-NMR (CDCl_3_) δ: 7.504–5.565 (m, 4H, H-2', H-6', *β*-H, *β*'-H), 7.208 (d, *J* = 8.4 Hz, 1H, H-6), 7.169 (s, 1H, H-2), 7.087 (d, *J* = 8.4 Hz, 1H, H-5), 6.921 (d, *J* = 9.0 Hz, 2H, H-3', H-5'), 3.871 (s, 3H, 3-OCH_3,_), 3.326 (t, *J* = 5.4 Hz, 4H, -CH_2_-N-CH_2_-), 3.088 (s, 4H, 3''-CH_2_, 4''-CH_2_), 2.632 (q, *J* = 7.2 Hz, 2H, -COC*H_2_*CH_3_), 1.687 (d, *J* = 4.8 Hz, 6H, -CH_2_-CH_2_-CH_2_-), 1.292 (t, *J* = 7.8 Hz, 3H, -COCH_2_C*H_3_*). ESI-MS *m/z*: 447.1 (M+1)^+^, calcd for C_28_H_31_NO_4_: 445.2.

*2-Methoxy-4-{(E)-[(E)-3-(4-morpholinobenzylidene)-2-oxocyclopentylidene]methyl}phenylpropionate*(**3c4**): Yellow powder, 65.4% yield, mp 186.1–189.4 °C. ^1^H-NMR (CDCl_3_) δ: 7.565 (s, 2H, H-2', H-6'), 7.260 (d, *J* = 16.2 Hz, 2H, *β*-H, *β*'-H), 7.211 (d, *J* = 7.8 Hz, 1H, H-6), 7.169 (s, 1H, H-2), 7.096 (d, *J* = 7.8 Hz, 1H, H-5), 6.951 (d, *J* = 9.0 Hz, 2H, H-3', H-5'), 3.874 (s, 3H, 3-OCH_3_), 3.284 (t, *J* = 4.8 Hz, 4H, -CH_2_-O-CH_2_-), 3.097 (s, 4H, -CH_2_-N-CH_2_-), 2.635 (q, *J* = 7.8 Hz, 2H, -COC*H_2_*CH_3_), 1.288 (t, *J* = 7.8 Hz, 3H, -COCH_2_C*H_3_*). ESI-MS *m/z*: 448.9 (M+1)^+^, calcd for C_27_H_29_NO_5_: 447.2.

*4-{(E)-[(E)-3-(2-fluorobenzylidene)-2-oxocyclopentylidene]methyl}-2-methoxyphenylisobutyrate* (**3d1**): Yellow powder, 48.9% yield, mp 148.6–151.2 °C. ^1^H-NMR (CDCl_3_) δ: 7.808 (s, 1H, *β*'-H), 7.595 (d, *J* = 7.2 Hz, 1H, H-4'), 7.572 (s, 1H, *β*-H), 7.340–7.385 (m, 1H, H-6'), 7.207 (t, *J* = 7.8 Hz, 2H, H-2, H-6), 7.121–7.161 (m, 2H, H-3', H-5'), 7.091 (d, *J* = 8.4 Hz, 1H, H-5), 3.867 (s, 3H, 3-OCH_3_), 3.063–3.107 (m, 4H, 3''-CH_2_, 4''-CH_2_), 2.833–2.879 (m, 1H, -COC*H*(CH_3_)_2_), 1.340 (d, *J* = 7.2 Hz, 6H, -COCH(C*H_3_*)_2_). ESI-MS *m/z*: 395.1 (M+1)^+^, calcd for C_24_H_23_FO_4_: 394.2.

*4-{(E)-[(E)-3-(4-Fluorobenzylidene)-2-oxocyclopentylidene]methyl}-2-methoxyphenylisobutyrate* (**3d2**): Yellow powder, 53.7% yield, mp 161.6–163.6 °C, HPLC purity 99.9% (condition A, r.t. 19.632 min). ^1^H-NMR (CDCl_3_) δ: 7.558–7.601 (m, 4H, H-2', H-6', *β*-H, *β*'-H), 7.214 (d, *J* = 8.4 Hz, 1H, H-6), 7.141 (t, *J* = 8.4 Hz, 3H, H-2, H-3', H-5'), 7.092 (d, *J* = 8.4 Hz, 1H, H-5), 3.867 (s, 3H, 3-OCH_3_), 3.104 (t, *J* = 6.6 Hz, 4H, 3''-CH_2_, 4''-CH_2_), 2.821–2.891 (m, 1H, -COC*H*(CH_3_)_2_), 1.340 (d, *J* = 7.2 Hz, 6H, -COCH(C*H_3_*)_2_). ESI-MS *m/z*: 394.8 (M+1)^+^, calcd for C_24_H_23_FO_4_: 394.2.

*2-Methoxy-4-{(E)-[(E)-3-(2-methoxybenzylidene)-2-oxocyclopentylidene]methyl}phenylisobutyrate* (**3d3**): Yellow powder, 78.5% yield, mp 149.9–153.1 °C, HPLC purity 96.827% (condition B, r.t. 14.967 min). ^1^H-NMR (CDCl_3_) δ: 8.026 (s, 1H, *β*'-H), 7.543 (t, *J* = 6.0 Hz, 2H, *β*-H, H-6'), 7.362 (t, *J* = 8.4 Hz, 1H, H-6), 7.206 (d, *J* = 6.6 Hz, 1H, H-4'), 7.159 (s, 1H, H-2), 7.081 (d, *J* = 8.4 Hz, 1H, H-5), 7.007 (t, *J* = 7.8 Hz, 1H, H-5'), 6.942 (d, *J* = 8.4 Hz, 1H, H-3'), 3.894 (s, 3H, 2'-OCH_3_), 3.864 (s, 3H, 3-OCH_3_), 3.068 (s, 4H, 3''-CH_2_, 4''-CH_2_), 2.831–2.877 (m, 1H, -COC*H*(CH_3_)_2_), 1.288 (t, *J* = 7.2 Hz, 6H, -COCH(C*H_3_*)_2_). ESI-MS *m/z*: 407.5 (M+1)^+^, calcd for C_25_H_26_O_5_: 406.2.

*2-Methoxy-4-{(E)-[(E)-3-(4-methoxybenzylidene)-2-oxocyclopentylidene]methyl}phenylisobutyrate* (**3d4**): Yellow powder, 64.5% yield, mp 150.9–154.8 °C, HPLC purity 88.228% (condition B, r.t. 15.206 min). ^1^H-NMR (CDCl_3_) δ: 7.581 (s, 2H, H-2', H-6'), 7.566 (s, 1H, *β*'-H), 7.535 (s, 1H, *β*-H), 7.213 (dd, *J* = 1.8, 8.4 Hz, 1H, H-6), 7.164 (s, 1H, H-2), 7.084 (d, *J* = 8.4 Hz, 1H, H-5), 6.972 (d, *J* = 9.0 Hz, 2H, H-3', H-5'), 3.864 (s, 6H, 3-OCH_3,_ 4'-OCH_3_), 3.101 (s, 4H, 3''-CH_2_, 4''-CH_2_), 2.832–2.878 (m, 1H, -COC*H*(CH_3_)_2_), 1.339 (t, *J* = 7.2 Hz, 6H, -COCH(C*H_3_*)_2_). ESI-MS *m/z*: 407.0 (M+1)^+^, calcd for C_25_H_26_O_5_: 406.2.

*4-{(E)-[(E)-3-(4-Ethoxybenzylidene)-2-oxocyclopentylidene]methyl}-2-methoxyphenylisobutyrate* (**3d5**): Yellow powder, 16.6% yield, mp 157.6–160.3 °C, HPLC purity 99.9% (condition A, r.t. 21.177 min). ^1^H-NMR (CDCl_3_) δ: 7.563 (d, *J* = 7.2 Hz, 2H, H-2', H-6'), 7.541 (d, *J* = 12.6 Hz, 2H, *β*-H, *β*'-H), 7.213 (dd,*J* = 1.8, 8.4 HZ, 1H, H-6), 7.169 (s, 1H, H-2), 7.096 (d, *J* = 8.4 Hz, 1H, H-5), 6.955 (d, *J* = 9.0 Hz, 2H, H-3', H-5'), 4.091 (q, *J* = 7.8 Hz, 2H, -OC*H_2_*CH_3_), 3.874 (s, 3H, 3-OCH_3_), 3.099 (s, 4H, 3''-CH_2_, 4''-CH_2_), 2.635 (q, *J* = 7.8 Hz, 1H, -COC*H*(CH_3_)_2_), 1.443 (t, *J* = 7.2 Hz, 3H, -OCH_2_C*H_3_*), 1.288 (t, *J* = 7.8 Hz, 6H, -COCH(C*H_3_*)_2_). ESI-MS *m/z*: 421.9 (M+1)^+^, calcd for C_26_H_28_O_5_: 420.2.

*4-{(E)-[(E)-3-(3,4-Dimethoxybenzylidene)-2-oxocyclopentylidene]methyl}-2-methoxyphenylisobutyrate* (**3d6**): Yellow powder, 24.8% yield, mp 152.4–154.1 °C, HPLC purity 97.988% (condition A, r.t. 22.933 min). ^1^H-NMR (CDCl_3_) δ: 7.553 (d, *J* = 14.4 Hz, 2H, *β*-H, *β*'-H), 7.238 (dd, *J* = 1.8, 8.4 Hz, 1H, H-6), 7.211 (dd, *J* = 1.8, 8.4 Hz, 1H, H-6'), 7.165 (s, 1H, H-2), 7.137 (d,*J* = 1.8 Hz, 1H, H-2'), 7.087 (d, *J* = 7.8 Hz, 1H, H-5), 6.946 (d, *J* = 8.4 Hz, 1H, H-5'), 3.939 (s, 6H, 3',4'-OCH_3_), 3.874 (s, 3H, 3-OCH_3,_), 3.118 (s, 4H, 3''-CH_2_, 4''-CH_2_), 2.832–2.878 (m, 1H, -COC*H*(CH_3_)_2_), 1.339 (d, *J* = 7.2 Hz, 6H, -COCH(C*H_3_*)_2_). ESI-MS *m/z*: 437.6 (M+1)^+^, calcd for C_26_H_28_O_6_: 436.2.

*4-{(E)-[(E)-3-(2,5-Dimethoxybenzylidene)-2-oxocyclopentylidene]methyl}-2-methoxyphenylisobutrate*(**3d7**): Yellow powder, 34.2% yield, mp 113.4–115.9 °C, HPLC purity 99.9% (condition A, r.t. 1.554 min). ^1^H-NMR (CDCl_3_) δ: 7.979 (s, 1H,*β*'-H), 7.544 (s, 1H, *β*-H), 7.204 (dd, *J* = 1.8, 8.4 Hz, 1H, H-6), 7.155 (d, *J* = 1.8 Hz, 1H, H-2), 7.091 (t, *J* = 8.4 Hz, 2H, H-6', H-5), 6.914 (dd, *J* = 1.8, 9.0 Hz, 1H, H-4'), 6.869 (d, *J* = 9.0 Hz, 1H, H-3'), 3.863 (s, 3H, 5'-OCH_3_), 3.849 (s, 3H, 2'-OCH_3_), 3.812 (s, 3H, 3-OCH_3_), 3.076 (s, 4H, 3''-CH_2_, 4''-CH_2_), 2.830–2.877 (m, 1H, -COC*H*(CH_3_)_2_), 1.338 (d, *J_1_*= 7.2 Hz, 6H, -COCH(C*H_3_*)_2_). ESI-MS *m/z*: 437.5 (M+1)^+^, calcd for C_26_H_28_O_6_: 436.2.

*2-Methoxy-4-{(E)-[(E)-2-oxo-3-(3,4,5-trimethoxybenzylidene)cyclopentylidene]methyl}phenylisobutyrate* (**3d8**): Yellow powder, 28.4% yield, mp 167.5–170.4 °C. ^1^H-NMR (CDCl_3_) δ: 7.543 (d, *J* = 16.8 Hz, 2H, *β*-H, *β*'-H), 7.212 (d, *J* = 6.6 Hz, 1H, H-6), 7.166 (s, 1H, H-2), 7.093 (d, *J* = 7.8 Hz, 1H, H-5), 6.850 (s, 2H, H-2', H-6'), 3.914 (s, 9H, 3',4',5'-OCH_3_), 3.865 (s, 3H, 3-OCH_3_), 3.130 (s, 4H, 3''-CH_2_, 4''-CH_2_), 2.833–2.891 (m, 1H, -COC*H*(CH_3_)_2_), 1.339 (d, *J* = 6.6 Hz, 6H, -COCH(C*H_3_*)_2_). ESI-MS *m/z*: 467.0 (M+1)^+^, calcd for C_27_H_30_O_7_: 466.2.

*2-{(E)-[(E)-3-(4-(Isobutyryloxy)-3-methoxybenzylidene)-2-oxocyclopentylidene]methyl}phenylisobutyrate* (**3d9**): Yellow powder, 54.5% yield, mp 128.4–131.6 °C. ^1^H-NMR (CDCl_3_) δ: 7.560 (s, 2H, H-3', H-6'), 7.446 (s, 1H, *β*'-H), 7.437 (s,1H, *β*-H), 7.312 (s, 1H, H-4'), 7.210 (dd, *J* = 1.8, 8.4 Hz, 1H, H-6), 7.162 (d, *J* = 1.2 Hz, 1H, H-2), 7.076–7.117 (m, 2H, H-5, H-5'), 3.863 (s, 3H, 3-OCH_3_), 3.109 (s, 4H, 3''-CH_2_, 4''-CH_2_), 2.788–2.894 (m, 2H, -COC*H*(CH_3_)_2_ × 2), 1.339 (t, *J* = 6.6 Hz, 12H, -COCH(C*H_3_*)_2_ × 2). ESI-MS *m/z*: 463.6 (M+1)^+^, calcd for C_28_H_30_O_6_: 462.2.

*4-{(E)-[(E)-3-(4-(isobutyryloxy)-3-methoxybenzylidene)-2-oxocyclopentylidene]methyl}phenylisobutyrate* (**3d10**): Yellow powder, 42.3% yield, mp 147.8–150.4 °C, HPLC purity 94.894% (condition A, r.t. 21.379 min). ^1^H-NMR (CDCl_3_) δ: 7.613 (d, *J* = 8.4 Hz, 2H, H-2', H-6'), 7.572 (d, *J* = 13.8 Hz, 2H, *β*-H, *β*'-H), 7.217 (dd, *J* = 1.8, 8.4 Hz, 1H, H-6), 7.166 (q, *J* = 1.8, 8.4 Hz, 3H, H-3', H-5', H-2), 7.092 (d, *J* = 8.4 Hz, 1H, H-5), 3.869 (s, 3H, 3-OCH_3_), 3.114 (s, 4H, 3''-CH_2_, 4''-CH_2_), 2.801–2.880 (m, 2H, -COC*H*(CH_3_)_2_ × 2), 1.337 (t, *J* = 7.2 Hz, 12H, -COCH(C*H_3_*)_2_ × 2). ESI-MS *m/z*: 463.0 (M+1)^+^, calcd for C_28_H_30_O_6_: 462.2.

*5-{(E)-[(E)-3-(4-(Isobutyryloxy)-3-methoxybenzylidene)-2-oxocyclopentylidene]methyl}-2-methoxyph-enylisobutyrate* (**3d11**): Yellow powder, 68.0% yield, mp 109.0–112.8 °C, HPLC purity 96.035% (condition B, r.t. 16.712 min). 7.530–7.539 (m, 2H, *β*-H, *β*'-H), 7.455 (dd, *J* = 1.8, 8.4 Hz, 1H, H-6'), 7.303 (d, *J* = 1.8 Hz, 1H, H-2), 7.207 (dd, *J* = 1.8, 8.4 Hz, 1H, H-6), 7.158 (d, *J* = 1.8 Hz, 1H, H-2'), 7.086 (d, *J* = 8.4 Hz, 1H, H-5), 7.013 (d, *J* = 8.4 Hz, 1H, H-5'), 3.870 (s, 3H, 4'-OCH_3_), 3.866 (s, 3H, 3-OCH_3_), 3.093 (s, 4H, 3''-CH_2_, 4''-CH_2_), 2.831–2.888 (m, 2H, -COC*H*(CH_3_)_2_ × 2), 1.343 (t, *J* = 7.2 Hz, 12H, -COCH(C*H_3_*)_2_ × 2). ESI-MS *m/z*: 494.4 (M+1)^+^, calcd for C_29_H_32_O_7_: 492.2.

*4-{(E)-[(E)-3-(4-(Isobutyryloxy)-3-methoxybenzylidene)-2-oxocyclopentylidene]methyl}-1,2-phenylene-bis(2-methylpropanoate)* (**3d12**): Yellow powder, 29.4% yield, mp 129.6–132.7 °C, HPLC purity 91.319% (condition B, r.t. 18.597 min). ^1^H-NMR (CDCl_3_) δ: 7.544 (d, *J* = 15.0 Hz, 2H, *β*-H, *β*'-H), 7.456 (dd, *J* = 1.8, 8.4 Hz, 1H, H-6'), 7.410 (d, *J* = 1.8 Hz, 1H, H-2), 7.247 (d, *J* = 8.4 Hz, 1H, H-5'), 7.209 (dd, *J* = 1.8, 8.4 Hz, 1H, H-6), 7.162 (s, 1H, H-2'), 7.092 (d, *J* = 8.4 Hz, 1H, H-5), 3.865 (s, 3H, 3-OCH_3_), 3.102 (s, 4H, 3''-CH_2_, 4''-CH_2_), 2.779–2.878 (m, 3H, -COC*H*(CH_3_)_2_ × 3), 1.311–1.345 (m, 18H, -COCH(C*H_3_*)_2_ × 3). ESI-MS *m/z*: 549.3 (M+1)^+^, calcd for C_32_H_36_O_8_: 548.2.

*4-{(E)-[(E)-3-(4-(Diethylamino)benzylidene)-2-oxocyclopentylidene]methyl}-2-methoxyphenylisobutyrate* (**3d13**): Purple powder, 40.4% yield, mp 126.1–128.7 °C, HPLC purity 99.800% (condition A, r.t. 22.872 min). ^1^H-NMR (CDCl_3_) δ: 7.512 (d, *J* = 8.4 Hz, 2H, H-2', H-6'), 7.260 (s, 2H, *β*-H, *β*'-H), 7.208 (dd, *J* = 1.8, 8.4 Hz, 1H, H-6), 7.166 (s, 1H, H-2), 7.072 (d, *J* = 7.8 Hz, 1H, H-5), 6.704 (d, *J* = 6.0 Hz, 2H, H-3', H-5'), 3.863 (s, 3H, 3-OCH_3_), 3.407–3.441 (q, *J* = 7.2 Hz, 4H, -CH_2_-N-CH_2_-), 3.085 (s, 4H, 3''-CH_2_, 4''-CH_2_), 2.830–2.876 (m, 1H, -COC*H*(CH_3_)_2_), 1.338 (d, *J* = 7.2 Hz, 6H, -COCH(C*H_3_*)_2_). ESI-MS *m/z*: 449.1 (M+1)^+^, calcd for C_28_H_33_NO_4_: 447.2.

*2-Methoxy-4-{(E)-[(E)-3-(4-morpholinobenzylidene)-2-oxocyclopentylidene]methyl}phenylisobutyrate* (**3d14**): Yellow powder, 57.6% yield, mp 177.7–180.2 °C. ^1^H-NMR (CDCl_3_) δ: 7.555 (d, *J* = 9.0 Hz, 2H, H-2', H-6'), 7.260 (s, 2H, *β*-H, *β*'-H), 7.209 (d, *J* = 1.8, 8.4 Hz, 1H, H-6), 7.163 (s, 1H, H-2), 7.081 (d, *J* = 8.4 Hz, 1H, H-5), 6.953 (d, *J* = 9.0 Hz, 2H, H-3', H-5'), 3.880 (t, *J* = 4.8 Hz, 4H, 3''-CH_2_, 4''-CH_2_), 3.864 (s, 3H, 3-OCH_3_), 3.285 (t, *J* = 4.8 Hz, 4H, -CH_2_-O-CH_2_-), 3.096 (s, 4H, -CH_2_-N-CH_2_-), 2.819–2.878 (m, 1H, -COC*H*(CH_3_)_2_), 1.339 (d, *J* = 7.2 Hz, 6H, -COCH(C*H_3_*)_2_). ESI-MS *m/z*: 462.6 (M+1)^+^, calcd for C_28_H_31_NO_5_: 461.2.

#### 3.2.4. Synthesis of **3c5**, **3c6**, **3d15**, and **3d16**

A solution of **1b** (0.010 mol) and cyclopentanone or hexanone (0.005 mol) in EtOH (12 mL) was stirred at room temperature, and HCl (gas) was bubbled into the mixture until the reaction was completed. Then the reaction mixture was cooled and poured into cold water (25 mL) to yield a colored precipitate, which was then reacted with propionyl or isobutyryl chloride in the presence of Et_3_N to prepare **3c5**, **3c6**, **3d15**, and **3d16**. The resulting crude product was purified by silica gel chromatography to give the target product as yellow solid.

*[(1E,4E)-3-Oxopenta-1,4-diene-1,5-diyl]bis(2-methoxy-4,1-phenylene) dipropionate* (**3c5**): Yellow powder, 26.9% yield, mp 138.8–141.6 °C, HPLC purity 98.707% (condition A, r.t. 8.474 min). ^1^H-NMR (CDCl_3_) δ: 7.694 (d, *J* = 15.6 Hz, 2H, *β*-H × 2), 7.222 (dd, *J* = 1.8, 8.4 Hz, 2H, H-6 × 2), 7.181 (d, *J* = 1.8 Hz, 2H, H-2 × 2), 7.079 (d, *J* = 8.4 Hz, 2H, H-5 × 2), 7.010 (d, *J* = 15.6 Hz, 2H, *α*-H × 2), 3.886 (s, 6H, 3-OCH_3_ × 2), 2.633 (q, *J* = 7.8 Hz, 4H, -COC*H_2_*CH_3_× 2), 1.286 (t, *J* = 7.8 Hz, 6H, -COCH_2_C*H_3_*× 2). ESI-MS *m/z*: 440.0 (M+1)^+^, calcd for C_25_H_26_O_7_: 438.2.

*[(1E,1'E)-(2-oxocyclohexane-1,3-diylidene)bis(methanylylidene)]bis(2-methoxy-4,1-phenylene)diprop-* *ionate* (**3c6**): Yellow powder, 10.4% yield, mp 135.7–138.9 °C, HPLC purity 91.7461% (condition A, r.t. 6.621 min). ^1^H-NMR (CDCl_3_) δ: 7.747 (s, 2H, *β*-H × 2), 7.066 (s, 4H, H-2 × 2, H-6 × 2), 7.040 (s, 2H, H-5 × 2), 3.849 (s, 6H, 3-OCH_3_ × 2), 2.928 (q, *J* = 7.2 Hz, 4H, 3''-CH_2_, 5''-CH_2_), 2.630 (q, *J* = 7.2 Hz, 4H, -COC*H_2_*CH_3_ × 2), 1.582 (s, 2H, 4''-CH_2_), 1.285 (t, *J* = 7.2 Hz, 6H, -COCH_2_C*H_3_* × 2). ESI-MS *m/z*: 479.9 (M+1)^+^, calcd for C_28_H_30_O_7_: 478.2.

*[(1E,4E)-3-Oxopenta-1,4-diene-1,5-diyl]bis(2-methoxy-4,1-phenylene)bis(2-methylpropanoate)* (**3d15**): Yellow powder, 8.4% yield, mp 122.9–125.8 °C, HPLC purity 95.8894% (condition A, r.t. 10.163 min). ^1^H-NMR (CDCl_3_) δ: 7.696 (d, *J* = 16.2 Hz, 2H, *β*-H × 2), 7.222 (dd, *J* = 1.8, 8.4 Hz, 2H, H-6 × 2), 7.179 (s, 2H, H-2 × 2), 7.067 (d, *J* = 7.8 Hz, 2H, H-5 × 2), 7.012 (d, *J* = 15.6 Hz, 2H, *α*-H × 2), 3.877 (s, 6H, 3-OCH_3_ × 2), 2.829–2.876 (m, 2H, -COC*H*(CH_3_)_2_ × 2), 1.337 (d, *J* = 7.2 Hz, 12H, -COCH(C*H_3_*)_2_ × 2). ESI-MS *m/z*: 467.2 (M+1)^+^, calcd for C_27_H_30_O_7_: 466. 2.

*[(1E,1'E)-(2-Oxocyclohexane-1,3-diylidene)bis(methanylylidene)]bis(2-methoxy-4,1-phenylene)bis(2-methylpropanoate)* (**3d16**): Yellow powder, 18.1% yield, mp 102.2–105 °C, HPLC purity 99.9% (condition A, r.t. 22.080 min). ^1^H-NMR (CDCl_3_) δ: 7.260 (s, 2H, *β*-H × 2), 7.048 (t, *J* = 6.6 Hz, 6H, H-2 × 2, H-6 × 2, H-5 × 2), 3.839 (s, 6H, 3-OCH_3_ × 2), 2.926 (t, *J* = 5.4 Hz, 4H, 3''-CH_2_, 5''-CH_2_), 2.814–2.872 (m, 2H, -COC*H*(CH_3_)_2_ × 2), 1.831 (t, *J* = 7.2 Hz, 2H, 4''-CH_2_), 1.334 (d, *J* = 7.2 Hz, 12H, -COCH(C*H_3_*)_2_ × 2). ESI-MS *m/z*: 508.0 (M+1)^+^, calcd for C_30_H_34_O_7_: 506.2.

### 3.3. Animals

Male C57BL/6 mice weighing 18–22 g were obtained from the Animal Center of Wenzhou Medical College (Wenzhou, China). The animals were housed under a constant room temperature with a 12:12 h light-dark cycle and fed with a standard rodent diet and water. The animals were acclimatized to the laboratory for at least 7 days before being used in the experiments. Protocols involving the use of animals were approved by the Wenzhou Medical College Animal Policy and Welfare Committee (Approval documents: 2012/APWC/0102).

### 3.4. Cell Treatment and ELISA

Mouse RAW264.7 macrophages were incubated in DMEM media (Gibco, Gaithersburg, MD, USA) replenished with 10% FBS, 100 U/mL penicillin, and 100 mg/mL streptomycin at 37 °C with 5% CO_2_. Cells were pretreated with compounds or vehicle control for 2 h, and then processed with LPS (0.5 μg/mL) for 22 h. After treatment, the culture media and cells were collected separately. The levels of TNF-α and IL-6 in the media were determined by ELISA using mouse TNF-α and mouse IL-6 ELISA Kits (eBioscience, San Diego, CA, USA). The total amount of the inflammatory factor in the media was normalized to the total protein amount of the viable cell pellets.

### 3.5. Descriptor Calculation and Selection

To obtain a QSAR model, compounds are often represented by molecular descriptors [28]. The molecular structures of all of the curcumin analogs were constructed with Maestro (Version 9.3 Schrödinger, LLC, New York, NY, USA). The full geometry optimization for the investigated molecules was performed with MOPAC2009 version 9.0.1. All the calculations were done based on the semi-empirical Parameterized model 6 (PM6) method [29]. The molecular descriptor computing was performed using MODEL (Molecular Descriptor Lab), a web-based server for computing structural and physicochemical features of compounds, according to the methods described in the literature [30]. The descriptors studied here contain the constitutional descriptors, physicochemical descriptors, topological descriptors, geometrical descriptors, charge (electronic) descriptors, and quantum chemistry descriptors. The optimized geometries of the molecules were uploaded to MODEL. After the calculation of the molecular descriptors, approximately 4,000 molecular descriptors based on the molecular 3D structure were obtained. The descriptors that remained constant for all molecules were eliminated, and pairs of variables with a correlation coefficient greater than 0.85 were classified as inter-correlated, and one in each correlated pair was deleted.

### 3.6. Multiple Linear Regression (MLR) Analysis

MLR analysis is a statistical technique that uses several explanatory variables to predict the outcome of a response variable [31]. The goal of multiple linear regressions (MLR) is to model the relationship between the explanatory and response variables. In our present study, MLR was performed using the program R, which is a powerful tool for statistical computing and graphics, to derive QSAR models. The biological data used in this study were the TNF-α- or IL-6-inhibitory rates compared to LPS group alone. Compounds with negative values were abandoned because of their pro-inflammatory activities. The inhibition rates against TNF-α and IL-6 release, IR_TNF-α_ and IR_IL-6_, respectively, were used as dependent variables in the linearization procedure. Subsequently, Stepwise Multiple Linear Regression (Stepwise-MLR) was used to select the significant descriptors. The most relevant descriptors were used as independent variables.

### 3.7. Validation of the Models

Validation of the linear models is required for testing the predictive ability and generalizing the methods by cross-validation. The leave-one-out procedure was employed. When a data point was removed from the analyzed set, the regression was recalculated, and then the predicted value for that point was compared to its actual value. This process was repeated until each datum had been omitted once, and then the sum of squares of these deletion residuals could be used to calculate q^2^, an equivalent statistic to R^2^.

### 3.8. In Vivo Study

The compounds were first dissolved with macrogol-15-hydroxystearate (a nonionic solubilizer for injection from BASF, Ludwigshafen, Germany) with or without medium chain triglycerides (MCT, from BASF,) in a water bath at 37 °C. The concentration of the compounds was 2 mg/mL. The concentration of solubilizer ranged from 5% to 10% and that of MCT ranged from 0.5% to 2% in the final solution. For the vehicle, the mixture of solubilizer and MCT was prepared at 10% and 2%, respectively. Male C57BL/6 mice weighing 18–22 g were pretreated with compounds (10 mg/kg) in a water solution by intravenous injection 15 min prior to the intraperitoneal injection of LPS (25 mg/kg). The control animals received a same volume (200 µL) of the vehicle. Body weight changes and mortalities were recorded for 7 days.

### 3.9. The Stability Analysis of Curcumin and Its Analogs by HPLC

The stability test of curcumin and its analogs were performed using a reverse phase HPLC (Agilent Technologies 1260 Infinity, Santa Clara, CA, USA). Briefly, 20 μL of 5 mM curcumin and its analogs (dissolved in methanol) were added to 980 μL of 0.1 M phosphate buffer (pH 7.4). Samples were incubated at 37 °C for indicated times. After incubation, 200 μL of mixtures were detected by HPLC with a mobile phase of methanol and water.

### 3.10. Cell Viability Assay

HL-7702 cells were used to determine the inhibitory effects of the curcumin analogs on cell growth using MTT assay. Cells were incubated with the test compounds for 24 h before the MTT assay. A fresh solution of MTT (5 mg/mL) prepared in phosphate buffer (pH = 7.4) was added to each single well. The plate was then incubated in a CO_2_ incubator for 3 h; cells were dissolved in 100 µL of DMSO, and then the optical density was read at 570 nm. Cell viability was determined as the percentage of untreated stimulated cells.

### 3.11. Statistical Analysis

All of the experiments were repeated more than three times. The results are shown as the mean ± SE. The statistics were analyzed using Student’s t-test in GraphPad Pro (GraphPad, San Diego, CA, USA). A *P*-value of less than 0.05 (*p* < 0.05) was considered significant.

## 4. Conclusions

In summary, we synthesized a series of curcumin analogs and evaluated their anti-inflammatory activities in LPS-stimulated RAW 264.7 macrophages. The majority of the compounds presented favorable anti-inflammatory activities. The SAR and QSAR analysis further demonstrated that the inhibition rate is highly related to the special substitutions, and may contribute to the further design of novel anti-inflammatory agents. Additionally, compounds **3b8**, **3b9**, **3c5**, **3c6**, **3d13** and **3d15** exhibited dose-dependent anti-inflammatory activities in macrophages. Two asymmetric analogs **3b8** and **3b9** were found to markedly decrease the LPS-induced lethality in a mouse model of sepsis. This result indicates that mono-carbonyl analogs of curcumin might serve as potential agents for the treatment of inflammatory diseases.

## Supplementary Materials

Supplementary materials can be accessed at: http://www.mdpi.com/1420-3049/19/6/7287/s1.
