# Supplementary material for: Synthesis and Evaluation of a Series of Novel Asymmetrical Curcumin Analogs for the Treatment of Inflammation
Source: Molecules. 2014 Jun 4;19(6):7287–307. doi: 10.3390/molecules19067287 (PMC6271832; doi:10.3390/molecules19067287)

Supplemental Materials

1. The Stabilities of Active Compounds Determined by HPLC and We Show the Initial Purity Here

1.1. RP-HPLC Spectrum of Compound 3b8: Purity 98.8875%

Figure S1. The purity of compound **3b8** is detected by HPLC.

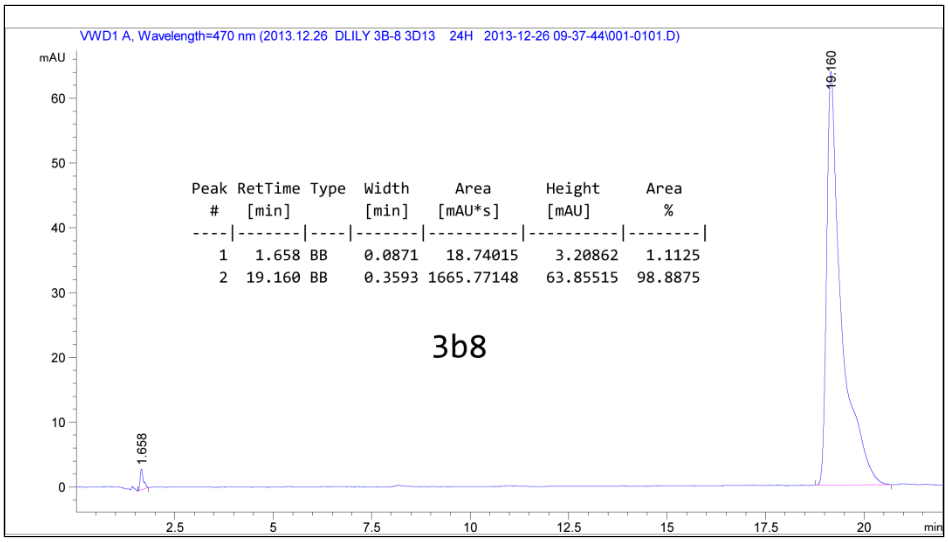

1.2. RP-HPLC Spectrum of Compound 3b9: Purity 91.8411%

Figure S2. The purity of compound **3b9** is detected by HPLC.

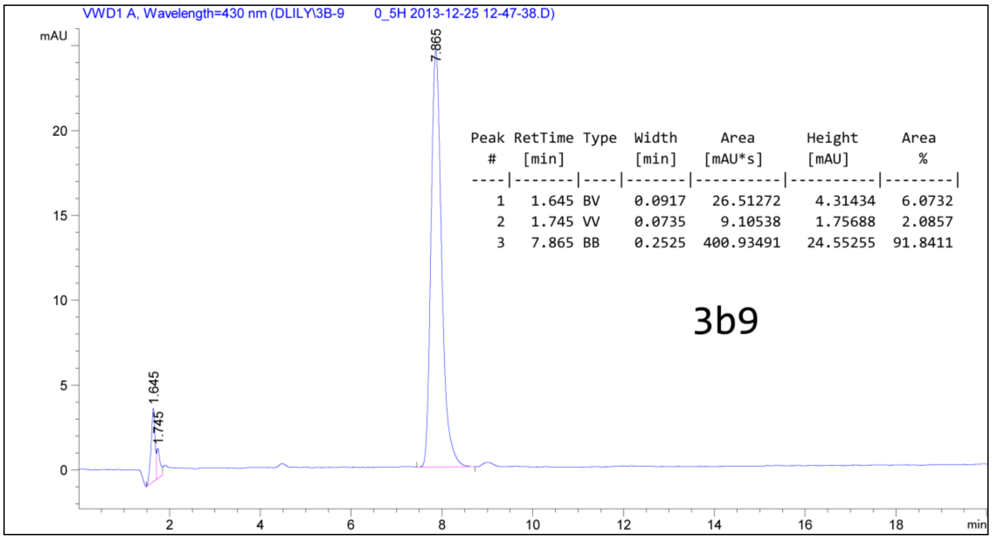

1.3. RP-HPLC Spectrum of Compound 3c5: Purity 98.7073%

Figure S3. The purity of compound **3c5** is detected by HPLC.

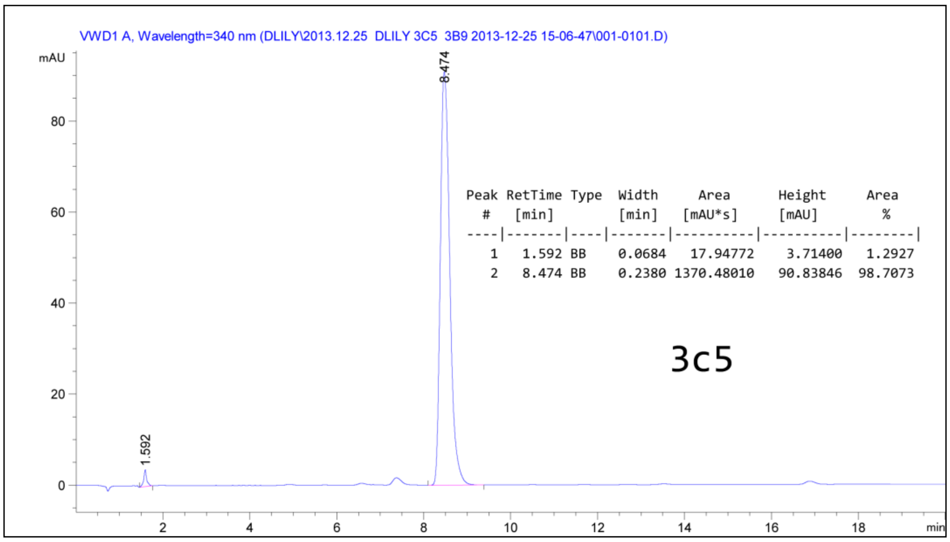

1.4. RP-HPLC Spectrum of Compound 3c6: Purity 91.7461%

Figure S4. The purity of compound **3c6** is detected by HPLC.

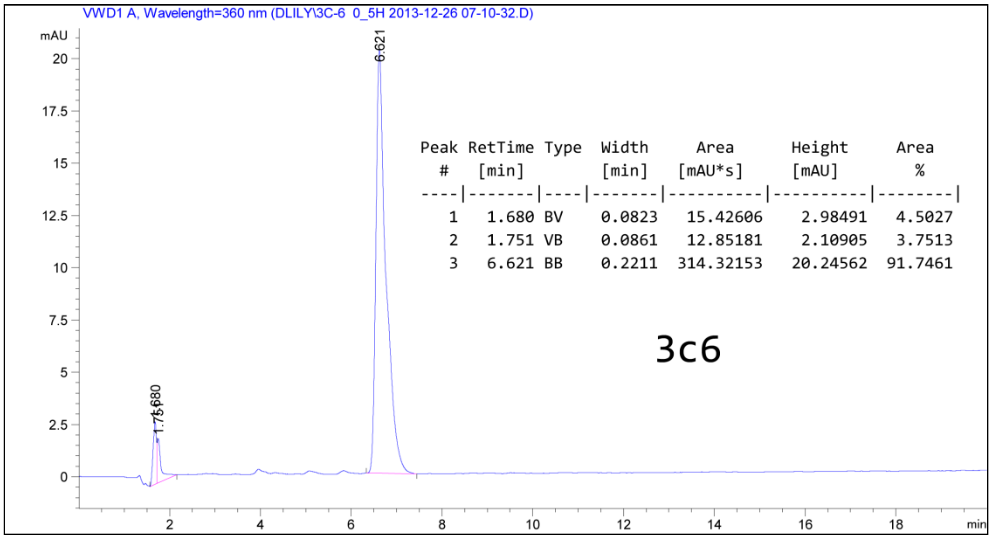

1.5. RP-HPLC Spectrum of Compound 3d13: Purity 99.7995%

Figure S5. The purity of compound **3d13** is detected by HPLC.

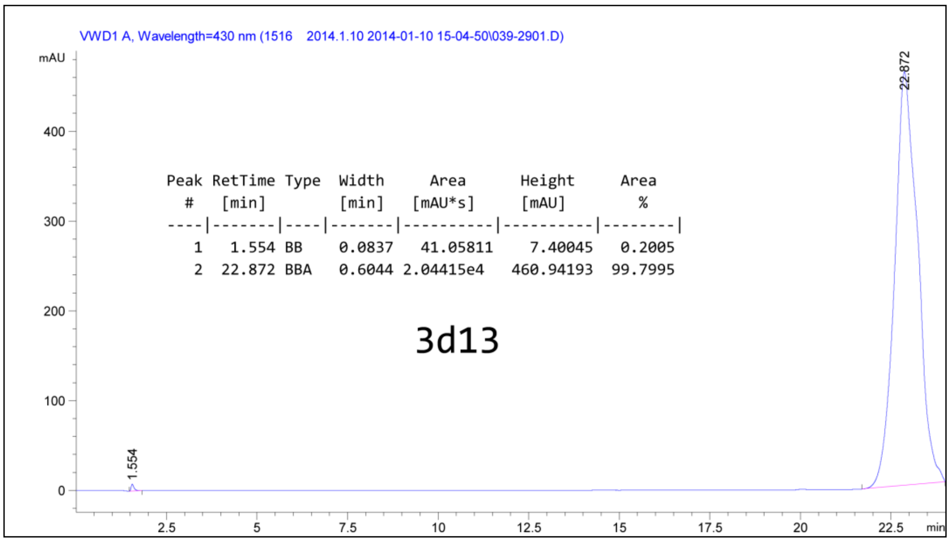

1.6. RP-HPLC Spectrum of Compound 3d15: Purity 95.8894%

Figure S6. The purity of compound **3d15** is detected by HPLC.

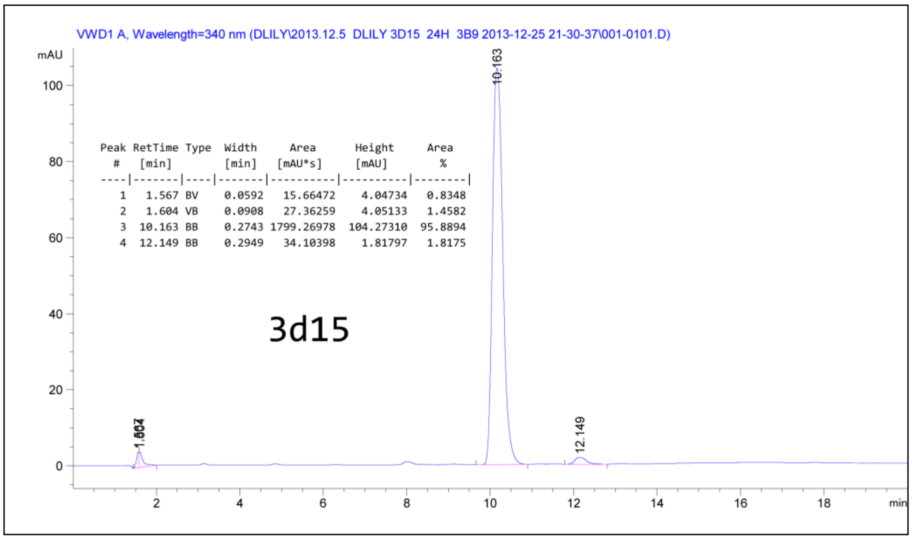

2. UV-Visible Absorption Spectra of Curcumin and Its Analogues

Absorbance readings were taken from 250 to 600 nm using a spectra Max M5 (Molecular Devices, Silicon Valley, California, USA). A stock solution of 1mM curcumin or its analogues (dissolved in DMSO) was prepared and diluted by phosphate buffer (pH 7.4) to a final concentration of 20 μM. In the experiments where degradation of curcumin was recorded, the absorption spectra were collected for over 25 min at 5 min intervals. The UV-visible absorbance spectrum was measured at 25 ℃ at varying time interval in a 1 cm path-length quartz cuvette.

Figure S7. UV-visible absorption spectra of curcumin, **3b8**, **3b9**, **3c5**, **3c6**, **3d13**, and **3d15** in phosphate buffer (pH 7.4), containing 5% DMSO.

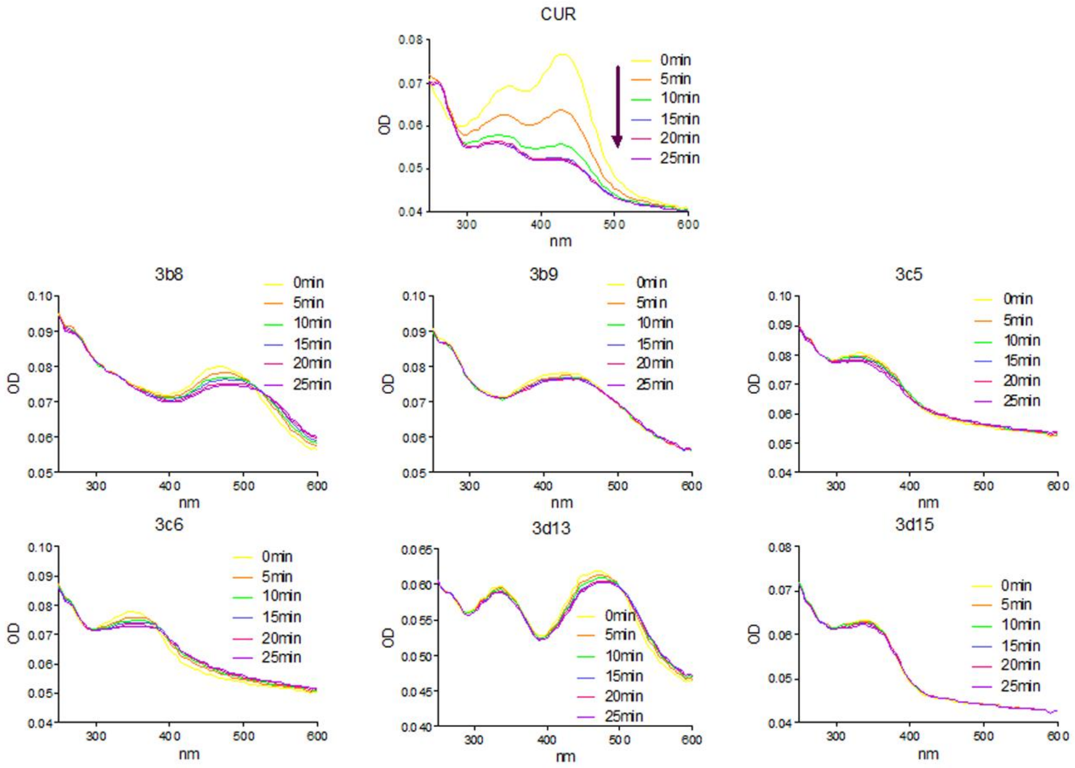

Supplement: Supplementary file 1 [file molecules-19-07287-s001.pdf]
